# peaks2utr: a robust Python tool for the annotation of 3′ UTRs

**DOI:** 10.1093/bioinformatics/btad112

**Published:** 2023-03-02

**Authors:** William Haese-Hill, Kathryn Crouch, Thomas D Otto

**Affiliations:** School of Infection & Immunity, MVLS, University of Glasgow, Glasgow G12 8TA, United Kingdom; School of Infection & Immunity, MVLS, University of Glasgow, Glasgow G12 8TA, United Kingdom; School of Infection & Immunity, MVLS, University of Glasgow, Glasgow G12 8TA, United Kingdom

## Abstract

**Summary:**

Annotation of nonmodel organisms is an open problem, especially the detection of untranslated regions (UTRs). Correct annotation of UTRs is crucial in transcriptomic analysis to accurately capture the expression of each gene yet is mostly overlooked in annotation pipelines. Here we present peaks2utr, an easy-to-use Python command line tool that uses the UTR enrichment of single-cell technologies, such as 10× Chromium, to accurately annotate 3′ UTRs for a given canonical annotation.

**Availability and implementation:**

peaks2utr is implemented in Python 3 (≥3.8). It is available via PyPI at https://pypi.org/project/peaks2utr and GitHub at https://github.com/haessar/peaks2utr. It is licensed under GNU GPLv3.

## 1 Introduction

Despite advances in genome assembly, little progress has been made in *ab initio* gene annotation, notwithstanding the vital role annotations play in the functional interpretation of data. The popular annotation pipeline MAKER2 is estimated to predict ∼80% of genes correctly (Holt and Yandell, 2011), highlighting the challenges of gene model annotation. Moreover, the annotation of UTR regions is generally ignored in most nonmodel organisms (Huang and Teeling, 2017). 3′ UTRs of messenger RNAs (mRNAs) are known to regulate mRNA-based processes, such as mRNA localization, mRNA stability, and translation (Mayr, 2019). Thus, understanding the expression and differential usage of 3′ UTRs is important in functional inference. There are several existing tools to predict UTRs including GETUTR (Kim et al. 2015), UTRme (Radío et al. 2018), ExUTR (Huang and Teeling, 2017), and F3UTER (Sethi et al. 2022). However, many of these tools are limited to specific organisms or require specific input that may not be available for nonmodel organisms (Supplementary Table S1). This partially explains why comprehensive genome annotation pipelines [e.g. Companion (Steinbiss et al. 2016)] neglect to offer annotation of 3′ UTRs. Fully annotated genomes are thus important to enable a more complete understanding of transcriptional regulation. Further, for comprehensive RNA-seq analysis, reads from the UTR should be captured in the analysis. This is especially true for methods where most reads map toward the end of UTRs, for example, 10× Chromium (Wang et al. 2021).

To improve this situation, we have developed peaks2utr to update 3′ UTR models in existing genome annotations using data from 10× Chromium sequencing runs, where signal is inherently concentrated at the distal ends of the 3′ or 5′ UTRs, allowing concise inference of UTR boundaries. The method described here addresses the use of 3′ 10× Chromium sequencing to improve 3′ UTR annotation. Its method is precise, as it uses only reads originating from the sense strand and seeks to apply a *s*oft-clipped *p*oly*A*-tail *t*runcation (SPAT) algorithm to read pileups. We will show that peaks2utr is more accurate, as well as easier to install and use, than existing tools and demonstrate the utility of its application in different datasets.

## 2 Workflow

peaks2utr (implemented in Python) is called from the command line and takes as input a GFF/GTF annotation file (with or without existing 3′ UTR annotations) and a BAM file containing aligned read data, as well as various optional parameters (see Supplementary Material).

BAM files are split into forward and reverse strands, and further into read groups, using pysam [a Python wrapper for htslib and SAMtools (Danecek et al. 2021)]. Now the SPAT algorithm is applied: reads containing soft-clipped bases and polyA-tails of a given length are identified, and their end bases are tallied as ‘truncation points’ (see Supplementary Material).

**Figure 1:**
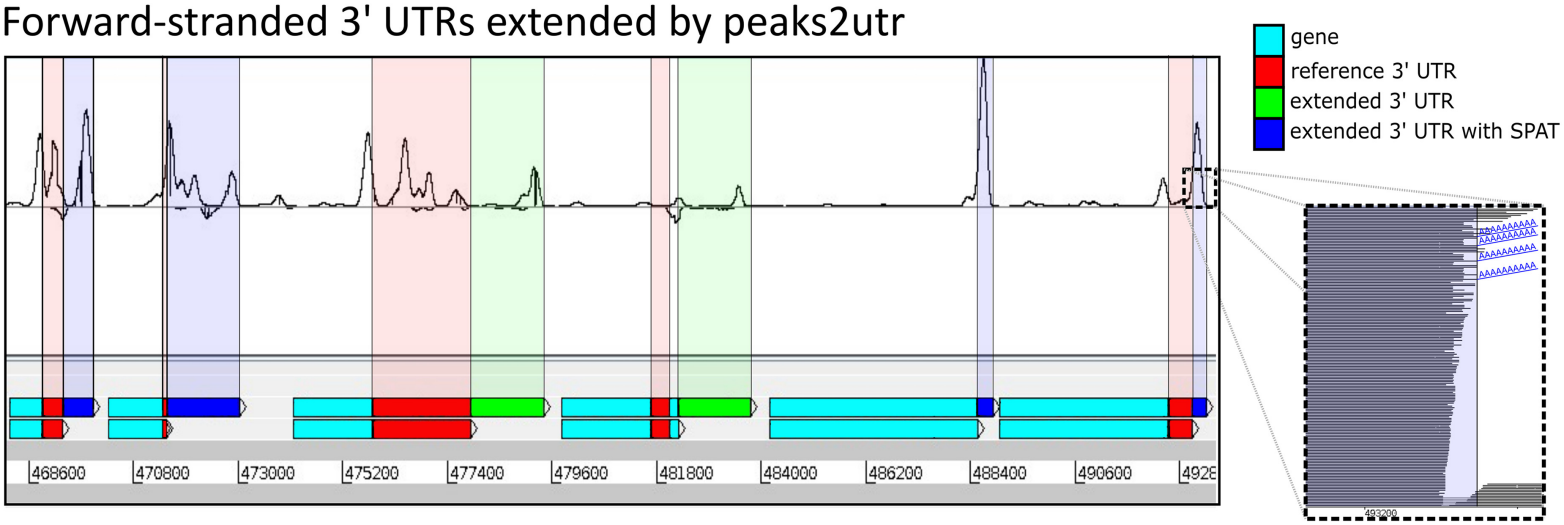
*Trypanosoma brucei* TREU927 genes with reference 3′ UTR. Top track shows the same genes with extension by peaks2utr, and how this coincides precisely with coverage peaks in 3′ region (as it is a 10× Chromium 3′ chemistry run); only the fourth gene from the left saw no extension—peaks2utr matched within a few bases. Inset shows magnification of mapped read stack, where SPAT has been applied to multiple reads with shared end base

Peaks are called from the BAM file for each strand using MACS2 (Zhang et al. 2008). Each peak is iterated over, and a search is performed for genes falling within a user-defined maximum distance of base pairs from the peak on the same strand. Subsequently, a peak is designated a 3′ UTR if it passes a set of criteria and truncated using SPAT if possible (Fig. 1, Supplementary Material).

## 3 Application

To understand the specificity and sensitivity of peaks2utr with a truth-set, we applied it to *Caenorhabditis elegans* reference chromosome I. We compared our results to GETUTR and UTRme. Overall, peaks2utr was able to find ∼60% of UTRs matching those in the canonical annotation (to within a 50 bp tolerance of the canonical UTR range), compared to ∼6% for the other two tools (see Supplementary Table S2). Further, peaks2utr did not overpredict the UTR length, which is in part due to the implementation of SPAT. A comparison of the three sets of new UTRs called by all tools revealed that 16 of them matched, with a number of these worthy candidates for future annotation (see Supplementary Fig. S3).

Next, we applied peaks2utr to the parasite *Trypanosoma brucei* (Briggs et al. 2021). Overall, out of 11703 annotated genes, 6179 have 3′ UTR annotations. By applying peaks2utr, we obtain in total 9888 3′ UTRs, an increase of ∼60% and covering ∼84% of available genes. Of these, 3709 are new, 5347 are altered, and the remaining 832 are unchanged. Figure 1 shows an example of seven annotated genes with 3′ UTRs extended by peaks2utr. For the 12.4 GB BAM file of ∼140 million mapped reads, and using 12 processors, runtime was ∼50 min; GETUTR was unable to run for a BAM file this large. To understand the impact of the changed annotation against the canonical reference annotation, we reran Cell Ranger 6.1.1 with the updated reference from peaks2utr. The peaks2utr annotation saw an improvement in genes captured per cell from 632 to 889 (∼33% increase), and UMI counts per cell from 797 to 1111 (∼39% increase), implying that a substantial signal is gained by using improved 3′ UTR annotations from peaks2utr.

## 4 Discussion

Here, we presented the tool peaks2utr that generates 3′ UTR annotations. It is easy to install, maintainable and includes unit testing. Leveraging the capture of the polyA-tail of the transcript allows us to map the transcript end to an unprecedented precision. In comparison with other tools, we assessed both ease of use and output. We had difficulty running several existing tools, as described in the Supplementary Material, highlighting the benefit of modern, easy-to-use tools like peaks2utr. We demonstrated that peaks2utr outperformed the existing tools that we were able to test. With the advent of scRNA-seq and its wide usage of nonmodel organisms, we recommend that scientists should run peaks2utr for their single-cell experiments to improve the capture of the signal (see Fig. 1). Further, it will help to improve the annotation of many species so far lacking 3′ UTR regions. Further development of peaks2utr will consider how to use long reads to improve 3′ UTR predictions.

## Supplementary Material

btad112_Supplementary_Data
