# Supplementary material for: peaks2utr: a robust Python tool for the annotation of 3′ UTRs
Source: Bioinformatics. 2023 Mar 2;39(3):btad112. doi: 10.1093/bioinformatics/btad112 (PMC10008064; doi:10.1093/bioinformatics/btad112)
Supplement: btad112_Supplementary_Data [file btad112_supplementary_data.pdf]

Supplementary Material

peaks2utr: a robust Python tool for the annotation of 3’ UTRs

William Haese-Hill, Kathryn Crouch, Thomas D. Otto

peaks2utr is implemented in Python 3 (≥ 3.8). It is available via PyPI at https://pypi.org/project/peaks2utr and GitHub at https://github.com/haessar/peaks2utr. It is licensed under GNU GPLv3. Here we describe some of the implementation and tests performed.

Method

Supplementary Figure 1 – Flowchart of peaks2utr processes

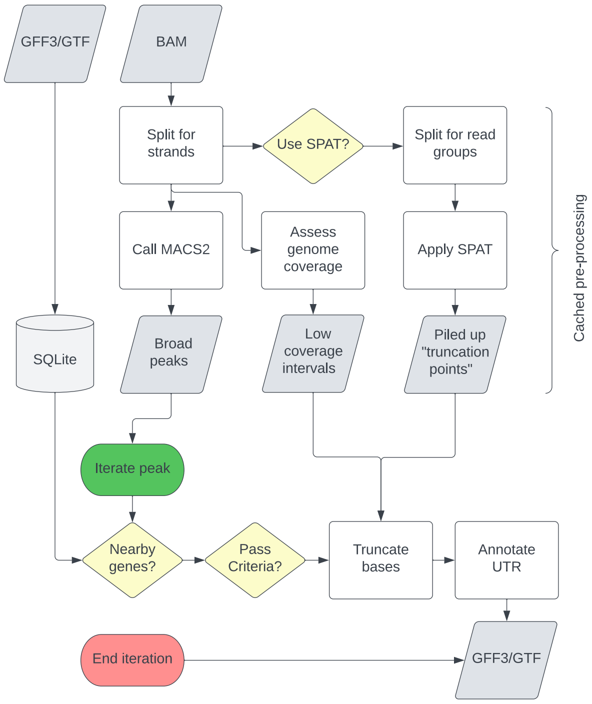

The overall workflow of peaks2utr can be seen in Supplementary Figure 1.

Several key aspects of the workflow are explained in greater detail in the following sections of this Supplementary Material:

- Peak to UTR assignment criteria
- UTR truncation methods (including soft-clipped polyA-tail truncation (SPAT) algorithm)
- Runtime enhancements

As indicated on the GitHub repository, peaks2utr can be installed simply with “pip install peaks2utr”.

Comments relevant to the implementation:

- peaks2utr does not generate *ab initio* models, it modifies the 3’ UTRs of existing gene models. The 3’ UTR annotation will be added as a “three_prime_UTR” feature type in the

annotation file, either as a child of the “mRNA” or “transcript” feature types when GFF or GTF output is desired, respectively.
- The 10x Chromium method captures polyadenylated transcripts and generates strand-specific reads. To minimise false positives and improve accuracy, antisense reads are discarded and only reads originating from the sense strand of each gene are considered for UTR-prediction.
- Although MACS2 (version 2.2.7.1), is optimised for ChIP-seq data, it was found to sufficiently predict regions of peaks likely to correspond to a UTR when tuned for “broad peaks”. In case of over-prediction, peaks2utr will automatically truncate to the edge of any interval containing zero reads (see below).

Current limitations of peaks2utr are as follows:

- It needs a genome and a basic gene annotation (it cannot find a UTR without a gene).
- It will return the longest gene model (so extending the UTR) and ignore potential alternative transcript ends.
- The SPAT algorithm implemented in peaks2utr is predicated on finding 3’ UTR termination by looking for polyadenylation, and peaks2utr has been developed and tested with 3’-biased data such as that generated by 10x Chromium. As such, this tool currently only predicts 3’ UTRs.

Peak to UTR assignment criteria

For each MACS2 peak, a set of five criteria are applied to determine whether it corresponds to a valid UTR. Supplementary Figure 2 shows visual examples of each of these criteria being applied.

i) If peak falls within a user-defined max-distance of base-pairs from the 3’-end of any gene, then it will be considered a potential UTR.
ii) If peak intersects a 3’ UTR that is already annotated in the input GFF file, either ignore, override or extend, depending on user preference.
iii) If peak range is a subset of a gene, then ignore.
iv) If peak intersects the 5’-end of the following gene on the same strand, any potential UTR will be truncated to the start base of that gene.
v) If peak is within max-distance (see (i)) of a gene, but also corresponds to 3’-end of the following gene, any potential UTR will only be assigned to the following gene.

Supplementary Figure 2 – Examples of peak assignment criteria; pink shaded regions correspond to MACS2 broad peak, green features are (potential) UTRs, teal features are genes.

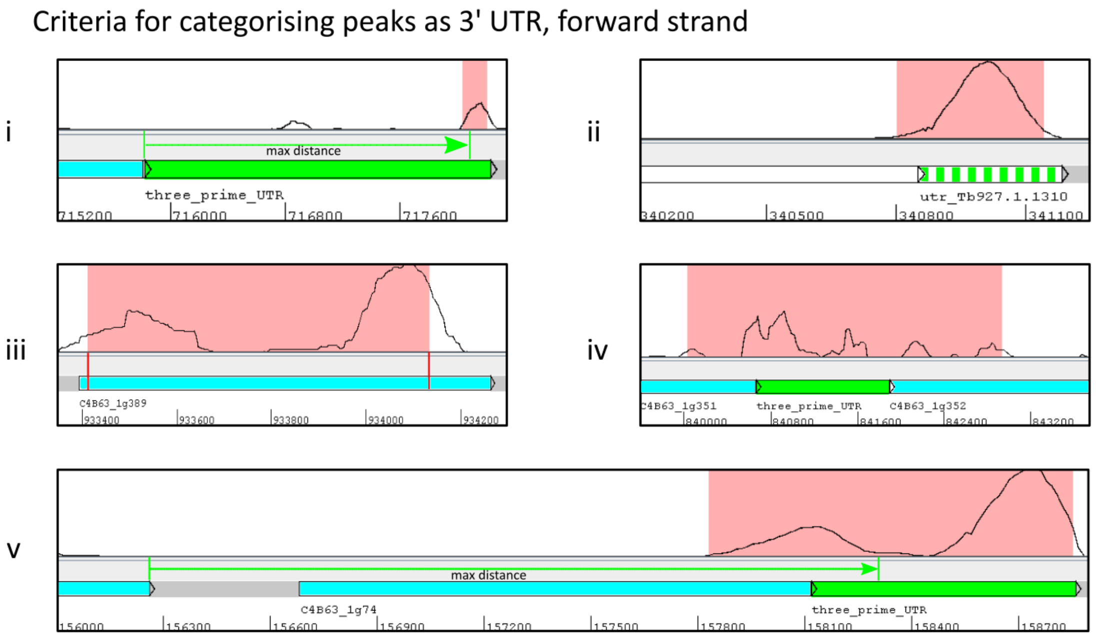

UTR truncation methods

During pre-processing, the following methods are applied to the input BAM file to allow precise truncation of any potential UTR feature that passes the criteria.

Filtering zero coverage intervals

Pybedtools (Dale, et al., 2011), a Python wrapper for BEDTools (Quinlan and Hall, 2010), is used to quantify read coverage across each stranded BAM file, and all intervals with zero coverage are cached. Any potential UTR that falls in one of these intervals is truncated to the edge of that interval. This compensates for the peaks called by MACS2 being too broad in certain circumstances, which would lead to overestimation of the 3’ UTR.

Soft-clipped polyA-tail truncation (SPAT) algorithm

Each read is parsed to determine whether it contains soft-clipped bases, and if so, whether the soft-clipped segment contains a polyA/T-tail with a minimum length specified by the user in an optional command line (CL) option --min-poly-tail (default 10). The mapped “end” bases of qualifying reads are tallied, and any that fall below a threshold specified by the user in an optional CL option --min-pileups (default 10) are filtered out.

The resulting set of bases are referred to as “truncation points”: If a truncation point falls within the range of a potential UTR, the UTR will be truncated to that base (or the outermost base in the case of multiple truncation points).

Runtime enhancements

A substantial factor in the overall runtime of peaks2utr is the pre-processing of files:

- gffutils builds a sqlite3 database to contain feature models for the input GFF/GTF file.

- pysam splits input BAM file into multiple batches for each strand to guarantee strand-independence of peaks, as the previously defined criteria are applied differently for forward and reverse. Each of these resulting BAM files are indexed.
- Zero read coverage intervals are stored as BED files.
- SPAT algorithm outputs truncation points to json files.
- MACS2 calls peaks on stranded-BAM files and outputs to broadPeak files.

To improve runtimes, peaks2utr harnesses multi-processing and caching of these pre-processed files (so that subsequent runs with tweaked parameters are significantly faster). The user can specify available processor cores with optional -p or –processors CL options. peaks2utr can utilise the inherent multi-processing functionality of samtools when calling any pysam function to manipulate BAM files, while gffutils and MACS2 pre-processing functions, being independent, are handled asynchronously.

SPAT pre-processing uses one available processor core for each BAM file split by strand/read group. MACS2 peaks for UTR annotation are batched depending on available processor cores.

The user must specify that the cache should be persisted with the –keep-cache CL option, to prevent any unwanted storage overhead.

As well as the three_prime_UTR annotations, a summary statistics text file is produced, informing the user about total UTR count, as well as number of peaks failing each criterion.

Overview of other UTR annotation tools

The following table outlines other 3' UTR annotation tools and any limitations we encountered when attempting to use them. In the following sections we describe our attempts to use these tools alongside peaks2utr for our test cases of a model and non-model organism (*Caenorhabditis elegans* and *Trypanosome brucei*, respectively).

Supplementary Table 1 – Limitations of UTR prediction tools.

	Language	Inputs	Limitations
peaks2utr	Python (v3)	Reference annotation (gtf), RNA-seq mapped reads (bam)	3'UTR calling
GETUTR	Python (v2)	Reference annotation (gtf), RNA-seq mapped reads (bam/bed)	Deprecated programming language; Memory consumption doesn't scale for larger bam files
ExUTR	Perl	Assembled transcripts (fasta), protein database (BLAST-compliant db/fasta), 3' UTR database (fasta)	Links to databases are no longer operational
UTRme	Python (v3)	Reference annotation (gff3), RNA-seq reads (fastq), Genome sequence (fasta), spliced-leader sequence	Intended for trypanosomes or organisms that use spliced leader trans-splicing
F3UTER	R	Reference annotation (gtf), aligned RNA-seq reads (bigwig), chr lengths, repeats data from RepeatMasker	Distributed as series of analysis pipeline scripts hardcoded for certain species

Caenorhabditis elegans application

We applied GETUTR (version 2.0.0) (Kim, et al., 2015), UTRme (Radío, et al., 2018) and peaks2utr to *C. elegans* as an example, as it contains a truth-set, through manual curation of UTRs, focusing on chromosome I. It was obtained from WormBase release WS283 (Davis, et al., 2022).

10x Chromium reads from a recent study (Packer, et al., 2019), with accession code GSE126954, were mapped against the full reference using 10x Genomics Cell Ranger 6.1.1 to generate the input

BAM file. Reads were acquired from 12 runs of SRA experiment SRX5411289. The resulting BAM file was 19 GB in size, which reduced to 3.3 GB when filtering for only chromosome I mapped reads, as used for the test.

GETUTR offers 3 smoothing algorithms to remove "noise" in the RNA-seq signal: Max.fit and Min.fit preference local maxima and minima, respectively, while PAVA uses weighted least-squares regression. Each UTR is output as a fragmented series of features with an assigned normalization scaling *s* between 0 and 1.

We ran GETUTR with PAVA smoothing and all features with $s < 0.95$ were removed to ensure highest quality and minimise overprediction of UTRs. To prevent GETUTR from crashing due to excessive virtual memory usage, we had to adapt the source code to prevent reading into memory of the entire samfile and instead use an iterator.

UTRme was configured with spliced-leader sequence *Nematoda 1*: GGTTTAATTACCCAAGTTTGAGGG (Bitar, et al., 2013), and otherwise run with default options.

peaks2utr was run with parameters --max-distance 2500, --override-utr, --min-pileups 4, and --min-poly-tail 5.

Supplementary Table 2 – Comparison of peaks2utr with GETUTR and UTRme on *C. elegans*. Reported are different cases of missed, new or extended UTR by both tools compared to the canonical annotation. Percentages are of total canonical UTR. peaks2utr outperforms both GETUTR and UTRme, especially by not overpredicting the UTR length and matching the range to within 50 bp.

	peaks2utr		GETUTR		UTRme	
	num	%	num	%	num	%
Total UTR	2278		2176		496	
Missed UTR	451	17.39	568	21.91	2119	81.72
**UTR matches (within 50 bp)**	**1578**	**60.86**	**155**	**5.98**	**157**	**6.05**
Shortened UTR*	117	4.51	513	19.78	121	4.67
Extended UTR*	211	8.14	133	5.13	19	0.73
**Too long UTR****	**236**	**9.10**	**1224**	**47.20**	**177**	**6.83**
Novel UTR†	136		151		22	
Sensitivity‡		85.5		64.2		25.1
Precision‡		45.3		21.1		36.4

* by more than 50 bp

** by more than 200 bp

† novel against WormBase annotations with gff3 source "WormBase". Some of these are annotated with "UTRome" source.

‡ metric from GFFCompare (Pertea and Pertea, 2020)

Supplementary Figure 3 – C. elegans protein coding gene WBGene00010291 (teal) with novel 3' UTR as predicted by peaks2utr (blue), GETUTR (yellow) and UTRme (fuchsia), one of 16 "novel" UTRs predicted by all three tools.*

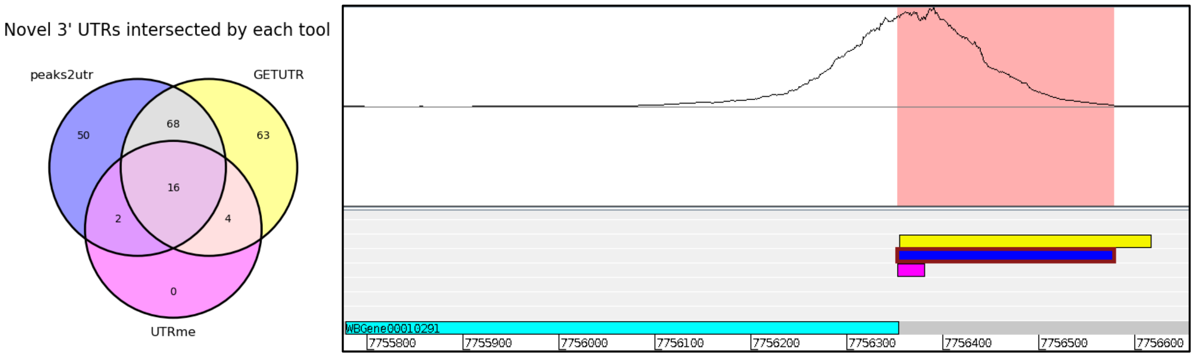

Supplementary Figure 4 – Weighted relationships between each tool for UTRs as expressed in Supplementary Table 2. The extent to which peaks2utr correctly matches UTR compared to other tools is evident, as is the comparative uniqueness between the results of GETUTR and peaks2utr. Most UTRs missed by peaks2utr were also missed by the other tools.

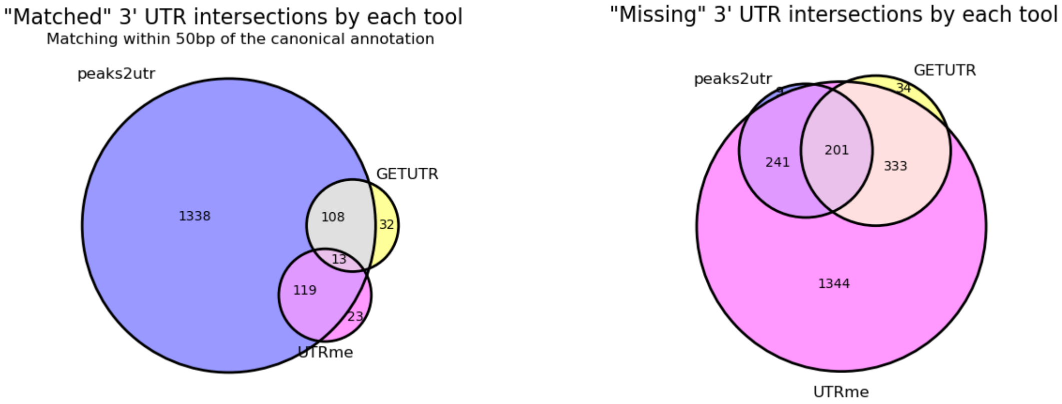

Trypanosome brucei application

To test the UTR tools on a non-model organism we chose the parasite *T. brucei*. It has polycistronic gene transcription and use splice leader sequence during the transcriptional process. The tool UTRme was especially built for this species complex.

For both biological replicates referred to in the main text, we ran 10x Genomics Cell Ranger 6.1.1 using 10x reads that were obtained from European Nucleotide Archive with study accession number PRJEB41744 and experiment accession number ERX5428965. The canonical annotation *Trypanosoma brucei brucei* TREU927 reference was obtained from TriTrypDB, release 43 (Aurrecoechea, et al., 2016).

peaks2utr was run with parameters --max-distance 2500, --override-utr. GETUTR was run with PAVA smoothing and filtering $s < 0.95$, as before. UTRme was run with default options.

Supplementary Figure 5 – A portion of T. brucei chromosome I forward strand (as used in Figure 1), with additional tracks for GETUTR (yellow) and UTRme (fuchsia) extended 3’ UTR. Unlike peaks2utr, neither tool was able to capture all 3’ UTR.

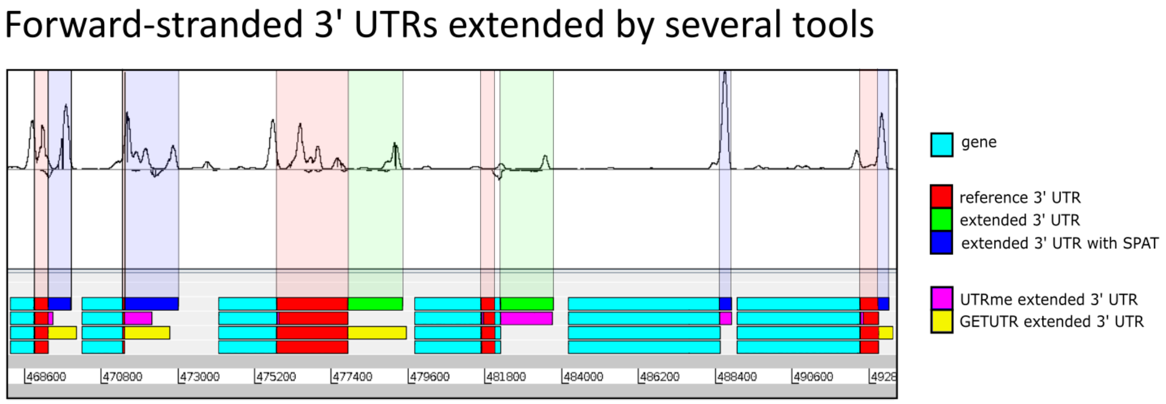

Failed attempts to compare peaks2utr with ExUTR and F3UTER

We were unable to produce a viable test case from ExUTR and F3UTER on either the *C. elegans* or *T. brucei* data.

Attempting to run F3UTER with either *C. elegans* (or *T. brucei)* resulted in failure. This reflects the very specialised nature of the pipeline scripts. We had a look into the source code and found that, even for the apparently generalised pipelines (such as https://github.com/sid-sethi/Generate-F3UTER-features) it is hardcoded for (going by the description) human and mouse data. This led us to use one of their prescribed examples for comparison (from https://github.com/sid-sethi/F3UTER/tree/master/Snakemake/F3uter_mouse_eval). The pipeline dependencies did not install on three different servers.

ExUTR requires several additional inputs such as files derived from 3’ UTR and swissprot databases. One of the input file links described in the installation instructions is no longer operational (http://utrdb.ba.itb.cnr.it/home/download). Our use-case in developing this tool is for non-model organisms where data available in these additional resources may be sparse or lacking entirely. For example, no hits were found in the suggested 3’ UTR database (in the ExUTR installation instructions) for some of the organisms we explore in this manuscript. While we were eventually able produce an output for *C. elegans*, it was in fasta format which proved difficult to reparse into the current annotation and compare with other tools.

References

Aurrecoechea, C.*, et al.* EuPathDB: the eukaryotic pathogen genomics database resource. *Nucleic Acids Research* 2016;45(D1):D581-D591.
Bitar, M.*, et al.* The spliced leader trans-splicing mechanism in different organisms: molecular details and possible biological roles. *Front Genet* 2013;4:199.
Dale, R.K., Pedersen, B.S. and Quinlan, A.R. Pybedtools: a flexible Python library for manipulating genomic datasets and annotations. *Bioinformatics* 2011;27(24):3423-3424.
Davis, P.*, et al.* WormBase in 2022—data, processes, and tools for analyzing Caenorhabditis elegans. *Genetics* 2022;220(4).
Kim, M., You, B.-H. and Nam, J.-W. Global estimation of the 3′ untranslated region landscape using RNA sequencing. *Methods* 2015;83:111-117.
Packer, J.S.*, et al.* A lineage-resolved molecular atlas of C. elegans embryogenesis at single-cell resolution. *Science* 2019;365(6459):eaax1971.

Pertea, G. and Pertea, M. GFF Utilities: GffRead and GffCompare [version 1; peer review: 3 approved]. *F1000Research* 2020;9(304).
Quinlan, A.R. and Hall, I.M. BEDTools: a flexible suite of utilities for comparing genomic features. *Bioinformatics* 2010;26(6):841-842.
Radío, S., *et al.* UTRme: A Scoring-Based Tool to Annotate Untranslated Regions in Trypanosomatid Genomes. *Frontiers in Genetics* 2018;9.
